# Synthetic data in medicine: Legal and ethical considerations for patient profiling

**DOI:** 10.1016/j.csbj.2025.05.026

**Published:** 2025-05-29

**Authors:** Maja Nisevic, Dusko Milojevic, Daniela Spajic

**Affiliations:** CiTiP KUL, Belgium

**Keywords:** Synthetic data, Patient profiling, Innovation and medicine, GDPR, AI Act, MDR, Biomedical ethics

## Abstract

Synthetic data is increasingly used in healthcare to facilitate privacy-preserving research, algorithm training, and patient profiling. By mimicking the statistical properties of real data without exposing identifiable information, synthetic data promises to resolve tensions between innovation and data protection. However, its legal and ethical implications remain insufficiently examined, particularly within the European Union (EU) regulatory landscape. This paper contributes to the emerging field of synthetic data governance by proposing a differentiated legal-ethical framework tailored to EU law. This paper follows a three-part taxonomy of synthetic data (fully synthetic, partially synthetic, and hybrid synthetic data) based on generation methods and identifiability risk. This taxonomy is situated within the broader context of the General Data Protection Regulation, the Artificial Intelligence Act, and the Medical Devices Regulation, clarifying when and how synthetic data may fall under EU regulatory scope. Focusing on patient profiling as a high-risk use case, the paper shows that while fully synthetic data may not constitute personal data, its downstream application in clinical or decision-making systems can still raise fairness, bias, and accountability concerns. The ethical analysis of profiling practices utilizing synthetic data is conducted through the lens of the four foundational biomedical principles: autonomy, beneficence, non-maleficence, and justice. The paper calls for sector-specific standards, generation quality benchmarks, and governance mechanisms aligning technical innovation with legal compliance and ethical integrity in digital health.

## Graphical Abstract

**Figure:**
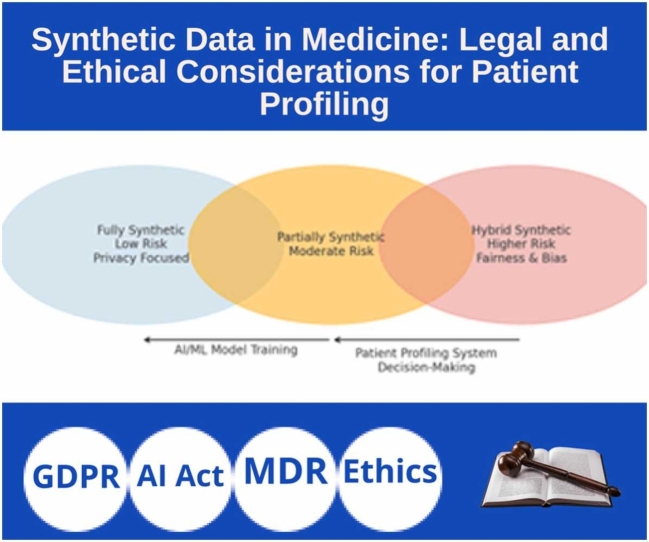

## 1 Introduction

The increasing application of artificial intelligence (AI), machine learning (ML), and Big Data in medicine has enabled the rapid evolution of diagnostic tools, therapeutic decision-making, and predictive analytics[1]. At the core of these innovations lies the practice of profiling (i.e., extracting patterns from data to make predictions or classifications about individuals or groups)[2]. Although it carries potential risks, profiling enables the personalization of medicine[3], optimization of care delivery, and identification of risk factors that might otherwise remain undetected in traditional clinical settings. However, using actual patient data for profiling also raises significant concerns about data privacy, consent, fairness, and accountability[4].

In response to these concerns, synthetic data has gained prominence as a privacy-preserving alternative[5]. In medicine, synthetic data is increasingly used for research, algorithm training, and system testing without infringing on patient confidentiality. This innovation promises to mitigate legal risks while enhancing the scalability and inclusiveness of patient profiling tools. Recent research indicates that *synthetic data analyses often closely mirror real-world datasets to the extent that even domain experts may struggle to differentiate between the two.* [6] However, synthetic data alone does not eliminate the ethical and legal complexities of profiling [7]. Although it reduces the risk of re-identification, it does not necessarily ensure fairness, transparency, or accuracy in profiling outcomes. Profiling practices raise challenging questions about responsibility, bias, and regulatory oversight, especially in automated or autonomic settings where machine learning models develop and apply profiles without human intervention [8], [9]. As Hildebrandt states, profiling represents *a new kind of knowledge production* (i.e., one that is probabilistic, often opaque, and capable of shaping reality rather than merely reflecting it) [10].

These concerns are reflected in recent EU legal instruments such as the General Data Protection Regulation (GDPR), the Artificial Intelligence Act (AI Act), and the Medical Devices Regulation (MDR), all of which aim to regulate data processing, risk assessment, and accountability in medicine and other high-impact domains. However, many gaps remain. For instance, the GDPR prohibits fully automated profiling that significantly affects individuals unless specific safeguards stipulated by the GDPR are in place [11]. However, it does not always clearly distinguish between the generation of profiles and their application [8], leading to uncertainty in contexts where synthetic or inferred data drive clinical predictions.

In this ethical, legal, and technological landscape, a deeper understanding of profiling (conceptually and in practice) and synthetic data is necessary to evaluate the promises of using synthetic data and its generation in patient profiling. This paper contributes meaningfully to the evolving field of synthetic data governance by providing a unique and nuanced legal and ethical analysis grounded in the EU regulatory framework. Instead of simply outlining technical definitions or offering broad overviews of regulations, we propose a detailed, risk-based taxonomy of synthetic data. This taxonomy distinguishes between fully, partially, and hybrid synthetic data, explicitly addressing the requirements set forth by the GDPR, the AI Act, and the MDR. Additionally, this paper uniquely combines legal and ethical perspectives to assess the implications of synthetic data in high-risk applications, demonstrating how regulatory compliance and normative principles intersect in the EU context. Our approach seeks to bridge a crucial gap between the quick pace of technical advancement and the intricate regulatory environment governing high-risk areas such as patient profiling in the EU. We recognize that many clinicians and researchers may already be familiar with some fundamental concepts related to synthetic data generation. However, we have intentionally included these elements to create a shared reference point and ground our legal and ethical analysis in patient profiling practices. What distinguishes this paper is its combination of technical discussion with a comprehensive legal and ethical interpretation that is particularly relevant within the EU regulatory landscape, an aspect not thoroughly explored in the existing literature.

### 1.1 Profiling as a technological phenomenon

While traditionally associated with direct marketing, profiling has emerged as a powerful and increasingly relevant tool in the healthcare sector, where it underpins a wide array of applications ranging from personalized medicine to predictive public health strategies.

Profiling is a complex and multidimensional practice encompassing the generation and application of structured representations of individuals or groups based on data correlations. Along with discovering correlations between data in databases used to identify and represent a human or non-human subject, individual or group, profiling practice also covers the application of profiles (i.e., sets of correlated data) to individuate and represent a subject [8], [10]. Profiling and its impact depend on the data's nature, the algorithms' transparency, and the context in which profiling outcomes are applied.

Following the typology of profiling, a distinction can be made between *human profiling* (conscious, heuristic categorization), *organic profiling* (natural pattern recognition by organisms), and *machine profiling* (executed by computational systems) [10].

Within machine profiling, a further distinction is made between *automated profiling* (i.e., algorithms assist human decision-making) and *autonomic profiling* (i.e., systems operate independently of human control) [8], [9].

Machine profiling, as a dominant method of inference and prediction nowadays, is directly correlated with the rise of Big Data Analytics ( i.e., Big Data, ML, and AI), which facilitates the discovery of patterns across vast and diverse datasets [8].

Technically speaking, profiling may be *individual or group-based*. Group profiling applies correlations derived from aggregate data to individual category members. In contrast, individual profiling assesses a specific subject based on their personal data. A further distinction exists between *direct profiling* (built from the subject's data) and *indirect profiling* (where one is assigned a profile based on data from similar individuals), which raises further questions about data accuracy and fairness [12].

From a technological perspective, profiles are not descriptive in a classical sense, mainly because they do not reveal absolute truths but probabilistic constructs derived through data aggregation and statistical modelling [8]. This probabilistic character introduces both legal and ethical concerns, mainly when using profiles to guide decisions about individuals without adequate context or oversight.

Profiling systems based on Big Data Analytics rely heavily on Knowledge Discovery in Databases (KDD), encompassing automated decision-making (ADM) across distinct profiling. KDD is a multistage process that includes data selection, cleansing, transformation, mining, and interpretation [13]. These technical processes form the backbone of modern AI-based profiling systems.

Ultimately, profiling is not merely a data analytics tool. It is both a knowledge-producing and normative practice. It creates meaning and guides action, whether in the commercial targeting of consumers or in medical decisions about diagnosis, treatment, or insurance eligibility. As such, individual rights and ethical principles must be critically assessed.

Despite rapid technological advancements, legal and policy frameworks frequently remain surpassed by the complexity of profiling systems, offering limited data processing regulation and the far-reaching implications of such systems' inferences [8].

### 1.2 Synthetic data: definitions, risks, and regulatory gaps

Scholars widely regard synthetic data as a potential solution for privacy-preserving data sharing, offering greater privacy with less compromise on utility than traditional anonymization techniques[5].

Additionally, synthetic datasets are increasingly considered an adequate safeguard against data leakage[14]. Synthetic data has recently garnered significant regulatory attention, notably following the publication of the U.S. Food and Drug Administration (FDA) Digital Health and AI Glossary, which defines *synthetic data as data that have been created artificially (e.g., through statistical modelling, computer simulation) so that new values and/or data elements are generated*[15]. This definition is gaining traction, especially in medical fields, as it offers a precise way to differentiate between synthetic and real-world datasets.

Although the FDA's definition has contributed to clarity for medical AI in the United States, it has not been widely accepted among scholars within the EU. In the EU, legal obligations under the GDPR do not depend solely on how data is generated but rather on whether the data can identify an individual (directly or indirectly) within a specific context.

While the FDA's definition provides a practical technical starting point, this paper argues that its inherent lack of granularity and primarily technical framing is not fully applicable to the legal and ethical complexities emerging under the GDPR, AI Act and MDR. In particular, it fails to consider the contextual nature of identifiability and the functional risks of data reuse in sensitive domains such as healthcare.

The FDA definition's broad nature does not specify the generation methods, source data, or acceptable statistical similarity or distortion levels, and overlooks crucial distinctions. Scholars like El Emam et al. suggest a more nuanced definition should also consider the privacy risk profile and utility-performance trade-offs inherent in different synthetic data generation approaches, rather than solely focusing on the generation method [16], [17]. High-fidelity synthetic data, while potentially offering greater analytical utility, may also carry a higher risk of re-identification than low-fidelity or highly generalized synthetic data, a crucial distinction not captured by the FDA's current framing.

Similarly, the FDA's technical focus does not adequately align with EU legal standards, especially the GDPR's emphasis on identifiability and purpose limitation. A synthetic record that technically contains *no direct* personal data might still lead to indirect identification, particularly when dealing with rare conditions or small populations, thus falling under GDPR's scope. The EU legal analysis necessitates a contextual understanding that extends beyond the data's mere *artificial* nature. This is further emphasized by the European Data Protection Supervisor (EDPS) [18], which highlights that *synthetic data generation implies a compromise between privacy and utility*. According to the EDPS, the more a synthetic dataset mimics the real data, the more utility it will have for analysts, but at the same time, the more it may reveal about real people, with risks to privacy and other human rights. Moreover, even if data is deemed *synthetic* under the FDA definition and thus theoretically non-identifiable, it can still perpetuate or amplify existing societal biases, leading to discriminatory outcomes, especially in sensitive areas like healthcare. An over-reliance on a purely technical definition risks overlooking these ethical implications, where the focus should extend beyond privacy protection to include the potential for real-world harm. While the FDA may consider resemblance to real individuals as coincidental, regulatory frameworks need to address potential harms arising from such coincidences, particularly in high-stakes applications.

Despite its potential, synthetic data generation falls short of being a foolproof solution. A significant concern is the risk of inadvertently *leaking sensitive information* when synthetic data too closely replicates real-world patterns [19], [20]. If not carefully generated, such data may retain identifiable characteristics, exposing individual information. This emphasizes the need for robust data generation techniques to protect privacy. Moreover, despite its growing prominence, particularly in facilitating timely access to health data for analysis and technological development, relatively few studies have specifically examined the application of synthetic data in medicine[21].

While the FDA’s definition serves as a valuable reference point, we propose a differentiated taxonomy to support more precise legal assessment and regulatory alignment in accordance with EU requirements. Legal scholars such as Wachter and Mittelstadt emphasize that the legal and ethical evaluation of data generation must be sensitive to both function and context of use[22]. This suggests that even when synthetic data is used solely for model training, the deployment of those models in real-world scenarios demands careful ethical and legal scrutiny.

Following the FDA's definition, which emphasizes artificial creation, *synthetic data* generated solely from statistical patterns and without direct inclusion of real-world data would, by definition, not contain personal data. However, the term *synthetic data* is also used to describe various classification approaches in the literature[23]. Synthetic data are commonly categorized into three broad types based on their relationship to the original dataset: fully synthetic, partially synthetic, and hybrid synthetic data[24].

*Fully synthetic data* is generated entirely from models that simulate real-world processes, containing no actual data points. This provides strong privacy protection but may compromise analytical utility due to the absence of real data[25]. While fully synthetic data offers strong privacy protection, any similarity to real individuals is generally considered coincidental and does not constitute reidentification. However, such resemblance may still raise ethical concerns in practice. *Partially synthetic data* incorporates a mixture of real and synthetic information (typically replacing only sensitive variables), which introduces a risk of re-identification[26]. *Hybrid synthetic data* combines real data structures with synthetic values, often using real datasets to guide generation processes[27].

Ultimately, this paper recognizes *synthetic data* in its various forms, enabling a more thorough examination of the legal and ethical challenges within the EU framework. The discussion moves beyond simply whether direct personal data is present to encompass the context of data use and the potential for harm[28].

Synthesis is the process of generating synthetic data. It aims to replicate the underlying patterns and structures of the original dataset. In principle, this should allow synthetic data to retain enough utility to be useful for research and analysis without revealing sensitive or identifiable information about real-world data subjects[29]. Data synthesis is subject to a balancing test between utility and anonymity[30]. The degree to which synthetic data is an accurate proxy for the original data is a measure of the utility of the method and the model. Anonymity (privacy), as derived from the definition of personal data in the GDPR, refers to the absence of identifiability. This implies that an individual cannot be identified, either directly or indirectly, through any identifiers or by combining various pieces of information. However, the real challenge lies in achieving a balance between utility and anonymity, as the synthesis involves a trade-off between privacy and utility. As the utility of a synthetic dataset increases, its level of anonymity often decreases, and vice versa[17]. High-utility synthetic data, which closely mirrors real-world data, is more likely to reveal identifiable patterns or characteristics that may compromise anonymity. Conversely, data with a stronger focus on privacy often sacrifices some of its analytical value, making it less useful for meaningful insights.

Understanding these fundamental differences is crucial for navigating the EU legal landscape. Table 1 compares each category's main legal and privacy factors in the taxonomy as mentioned above[31].

**Table 1:** Key Distinctions Between Fully Synthetic, Partially Synthetic, and Hybrid Synthetic Data in the EU Legal Context.

Feature	Fully Synthetic Data	Partially Synthetic Data	Hybrid Synthetic Data
Contains Real Data?	**X** No (artificially generated from statistical models/patterns)	**✓** Yes (some real variables retained, others synthesized)	**✓** Yes (real data structures combined with synthetic values)
GDPR Applicability?	⚠️ Likely not directly, but the context of use is crucial.	**✓** Yes (due to the presence of real/identifiable data)	**✓** Yes (due to the presence of real/identifiable data)
Re-identification Risk?	⬇️ Low	➡️ Moderate	⬆️ High
Utility for Analysis?	⬇️ Potentially Lower (depends on model fidelity and utility)	➡️ Moderate to High (balances privacy)	⬆️ High (mirrors real-world structures)
Ethical Considerations?	Transparency of the generation process, potential for misuse	Re-identification risks, transparency, fairness	Re-identification risks, transparency, fairness, and accountability

As illustrated in Table 1, partially and hybrid synthetic data present more significant re-identification risks than fully synthetic data, which has implications for legal compliance in the EU.

### 1.3 Patient profiling and synthetic data

In recent years, patient profiling has evolved beyond simple stratification models toward highly dynamic, data-driven systems powered by artificial intelligence and, increasingly, synthetic data. A growing body of interdisciplinary research emphasizes the role of patient profiling as a core component of modern, personalized medical treatment. As Rosano et al [32]. observe in the context of heart failure, traditional *one-size-fits-all* therapeutic models are being replaced by phenotype-guided profiling systems, which adapt treatment to a patient’s hemodynamic and renal characteristics. The authors stress that adjusting Guideline-Directed Management and Therapy (GDMT) to the patient’s hemodynamic profile may allow for a better and more comprehensive therapy for each patient than the more traditional hierarchical approach. This emphasis on tailoring care through profiling extends across domains and is becoming foundational to precision medicine.

In orthopedics, de Windt et al [33]. demonstrated, based on profiling, that patients under the age of 30 and those treated for cartilage defects within 24 months of injury experienced significantly better clinical outcomes measured by the Knee injury and Osteoarthritis Outcome Score (KOOS). They concluded that patient age, lesion location, and time since injury significantly influence treatment success and should inform a more detailed estimation of the patient’s prognosis and a patient-specific treatment strategy.

Similarly, in mental health, Delgadillo et al [34]. introduced the Leeds Risk Index (LRI), a profiling tool used to stratify patients based on variables such as functional impairment, disability, and treatment expectancy. Their findings confirmed that different people respond differently to therapy and that risk-based profiling can effectively predict therapy dropout and treatment resistance, thereby supporting individualized therapeutic intensities.

The utility of profiling is further evidenced in physiological modelling. Foley et al [35]. developed individualized thrombin generation profiles to assess coagulation risk in patient subgroups, including individuals with hemophilia and pregnancy-related hemostatic changes. Their method provided a unique level of resolving power concerning individual differences, offering a promising pathway for dynamic, multivariate risk assessment. These profiling techniques rely on integrating multiple data points, often longitudinal in nature, to model disease trajectories or treatment responses at the individual level.

Beyond empirical data, synthetic data generation has emerged as a novel means of enabling patient profiling while upholding privacy standards. D’Amico et al [36]. demonstrated that synthetic patient cohorts generated using conditional generative adversarial networks (cGANs) were able to recapitulate all clinical endpoints of the original study and identify molecular risk categories with the same stratification accuracy as real-world cohorts. Such synthetic profiling preserves the statistical structure of clinical and genomic datasets without exposing actual patient identities. Reflecting this, Adam [37] observed that the use of synthetic data makes it much harder to trace and identify those who volunteered their data, thereby reducing legal risk while preserving analytical utility.

Collectively considering the above-mentioned findings, they illustrate that patient profiling is not only a clinical and predictive tool but also an ethical and methodological challenge, particularly when applied to sensitive health data. *Accordingly, patient profiling is the systematic process of identifying and categorizing individuals based on a combination of demographic, physiological, clinical, behavioural, or genomic attributes in order to inform diagnostic, prognostic, and therapeutic decisions.* It serves as a foundational pillar of precision medicine and is increasingly supported by AI. Additionally, synthetic data offers a compelling solution to this dilemma by enabling privacy-preserving modelling of diverse patient trajectories. When governed by rigorous validation, fairness, and transparency standards, patient profiling based on synthetic data generation can replicate real-world clinical logic without compromising individual privacy.

While patient profiling based on synthetic data offers significant potential for advancing privacy-preserving precision medicine, its legal and ethical implications depend heavily on how the data is generated and applied. When synthetic data is entirely artificial, generated without reference to real individuals and devoid of any direct or indirect identifiers (i.e., fully synthetic data), it generally falls outside the scope of the GDPR, as it does not pertain to an identifiable person. In such cases, profiling practices do not trigger personal data protection obligations under EU law. Profiling systems based on fully synthetic data can replicate real-world clinical logic without compromising individual privacy, provided they are governed by rigorous standards of validation, fairness, and transparency. However, legal and ethical challenges may still arise when models trained on synthetic data are applied to real individuals in clinical contexts. Even if the training data is non-identifiable, the use of such models in real-world decision-making may reintroduce concerns related to fairness, bias, accountability, and transparency. In particular, questions remain regarding how profiling systems, especially those classified as high-risk under the EU AI Act, should be evaluated when trained on synthetic data but used to guide decisions affecting individuals or groups. While synthetic data may mitigate privacy risks at the point of generation, its use may give rise to a host of challenges that require ongoing legal scrutiny and ethical oversight to ensure accuracy, prevent the perpetuation of bias, and uphold core data protection principles within the evolving EU regulatory landscape.

## 2 Legal and ethical implications of patient profiling based on synthetic data

Synthetic data generation represents one of the most promising privacy-preserving techniques in modern data-driven medical research. These methods aim to produce artificial datasets that preserve the statistical properties of real data, offering a way to share information and train machine learning models without exposing sensitive personal information. Synthetic data generation aims to mimic real-world data while maintaining confidentiality and privacy. While various techniques are employed for synthetic data generation, Generative Adversarial Networks (GANs) and Variational AutoEncoders (VAEs) are the most prevalent[38].

The objective of synthetic data generation is to preserve statistical properties while ensuring the generated data is sufficiently different from the original data in terms of its private features[39]. The statistical conclusions drawn from synthetic data should closely reflect those from real data, allowing it to retain utility for research and analysis without compromising privacy.

Synthetic data enables researchers to simulate patient outcomes, model diseases, and refine treatment strategies without directly relying on identifiable health records. This innovation promises to mitigate legal risks while enhancing the scalability and inclusiveness of patient profiling tools. Despite these advantages, synthetic data also presents profound legal and ethical challenges, particularly in highly regulated domains like medicine. One of the central issues is achieving and verifying *proper anonymization.* According to Recital 26 of the GDPR, data is considered anonymous when individuals are no longer identifiable, either directly or indirectly. However, in practice, defining the technical threshold for anonymity remains contested. This is especially problematic for health-related data, such as genetic information, where the inherent identifiability of DNA renders complete anonymization nearly impossible [40]. These concerns highlight the need for both *legal clarity and technical standards* to define when synthetic data meets GDPR requirements.

Moreover, the lifecycle of synthetic data introduces further risks. Even when data is designed to be anonymous at the point of generation, subsequent sharing, aggregation, or external linkage can lead to *re-identification*, mainly when robust comparative datasets are available. This risk is magnified in medical contexts, where patient data is especially sensitive[41]. For example, when synthetic health records are shared with third parties, their integration with other datasets can inadvertently expose private patient details unless rigorous safeguards are in place.

Another significant challenge involves *bias and representativeness*. Synthetic data is typically generated from real datasets, meaning structural biases embedded in the source material, such as underrepresenting certain demographic groups, may be replicated and even amplified. In the medical domain, this can result in inequitable outcomes, such as misdiagnosis or ineffective treatment recommendations for already marginalized populations. As Chen, Joshi, and Ghassemi [42] cautioned, synthetic datasets trained on biased inputs can reinforce existing disparities, particularly in high-stakes contexts like clinical decision-making.

These risks highlight the need for *continuous oversight and robust evaluation frameworks.* As emphasized in recent studies[43], ensuring that *synthetic data remains anonymous, unbiased, and legally compliant* throughout its lifecycle requires the integration of ethical design principles, rigorous technical validation, and ongoing legal supervision. Additionally, some scholars point out that even if synthetic data closely matches the statistical distribution of real data, it still fails to account for the dynamic nature of the real world. Real data is continually evolving as new information emerges, behaviours change, and external factors influence outcomes. In contrast, synthetic data is effectively a "snapshot in time," representing the state of the original data at the moment it was created[44].

While the GDPR offers high-level privacy protections, it lacks specific technical standards for anonymization in complex domains such as synthetic data generation. This gap has been acknowledged in academic literature [45] and policy discussions, including the European Commission's 2022 Conference on Synthetic Data[46], which called for harmonized legal and technical standards for synthetic data use across sectors.

While the European Union lacks specific guidelines on synthetic data, valuable insights into the ethical handling of such data can be drawn from guidelines issued by bodies like the UK Statistics Authority and the Office for National Statistics (ONS)[47] and International organisations' policy documents, such as the OECD[48]. These guidelines address essential legal and ethical considerations, such as confidentiality and the risks of data disclosure. They provide a crucial framework for responsible data use, helping researchers navigate privacy and data protection issues. Scholars such as Wachter and Mittelstadt [49] argue for an *interdisciplinary approach*, emphasizing that data protection law should not be treated merely as a constraint but as a core component of ethical AI development. This perspective is critical in medicine, where ethical obligations (such as respecting patient autonomy and ensuring fairness) must remain at the forefront of innovation.

More recently, Giuffrè and Shung [50] have emphasized that the promise of synthetic data in medical research must be balanced against concerns of inclusivity and equity. They advocate for *real-time bias monitoring and corrective mechanisms* to prevent harm to vulnerable populations. Similarly, Hardjono et al [51]. call for regulatory frameworks incorporating *transparency, traceability, and accountability* throughout the synthetic data lifecycle. They suggest developing third-party auditing and certification mechanisms to verify whether synthetic datasets meet ethical and legal standards, including anonymity, data governance, and intended-use alignment.

Concerns have also been raised about the *scientific validity* of evidence derived from synthetic data, mainly when used in clinical settings. Binns [52] points out that inaccuracies or distortions in synthetic datasets, mainly from biased or incomplete source data, can compromise medical conclusions. He argues that data generation processes should be documented transparently and include mechanisms to assess and correct bias, mainly to protect underrepresented groups from misrepresentation or harm.

In light of these challenges, synthetic data's ethical and legal assessment must move beyond formal compliance to include *ongoing monitoring, participatory governance, and procedural transparency*. Only by embedding these safeguards can synthetic data's potential in profiling practices be fully realized in a way that enhances, rather than undermines, trust in digital health innovation.

### 2.1 The EU regulatory landscape

The General Data Protection Regulation (GDPR) represents one of the most robust legal frameworks for protecting personal data within the EU[53]. Its relevance is particularly essential in medicine, where the data involved is often personal and sensitive, including information about health, genetics, and biometric identifiers [54].

Synthetic data in patient profiling raises novel questions regarding data protection rights, the regulation of profiling and ADM [55], and the specific protection afforded to children's data.

If synthetic data is fully anonymized, it falls outside the scope of the [56]. However, as discussed in the previous section, determining whether synthetic data is genuinely anonymous remains challenging. If the generation process is derived from small or unique datasets, especially those related to children or genetic data, re-identification risks may persist, particularly when combined with external datasets [31], [32].

Synthetic data that retains patterns that are traceable back to individuals, especially when generated from rare conditions or small pediatric populations, may still be considered *personal data* under the GDPR. This blurs the legal boundary between anonymized data and pseudonymized or indirectly identifiable data, which remains subject to GDPR protections.

One of the central provisions relevant to synthetic data use in profiling is Article 22 of GDPR, which restricts fully automated decision-making, including profiling, when it produces legal or similarly significant effects on individuals [7], [8], [9]. In a medical context, such effects may include diagnostic outcomes, treatment eligibility, or prioritization for clinical trials.

Even when synthetic data is used to *develop a profiling system*, deploying that system on real individuals triggers Article 22 if decisions are made without meaningful human involvement. This means that profiling tools trained on synthetic data are not exempt from GDPR obligations if they are later used in automated clinical systems.

The GDPR recognizes exceptions to ADM and profiling when it is based on explicit consent, contract, or authorized by Union or Member State law [7], [8], [9]. Additionally, the GDPR requires data subjects to be informed about the existence of ADM, profiling, the logic involved, and the significance and envisaged consequences [57], [58]. Transparency is crucial, even if the model itself was developed using synthetic data. Whether based on real or synthetic inputs, profiling systems must offer explainability, especially in medical contexts where life-altering outcomes may result from AI-driven classifications [59], [60], [61].

While synthetic data supports the GDPR principles of data minimization and privacy by design [62], its use must still conform to purpose limitation under Article 5(1)(b). This means that the purposes for generating synthetic data must be clearly defined, and the data cannot be repurposed for unrelated objectives without further legal justification.

In medical research, synthetic datasets often represent artificially generated patient journeys. However, if they are structurally close to real individuals and if used to train systems that later act on real patients, the ethical obligation to *evaluate representativeness and fairness* becomes central, particularly where synthetic profiles are later applied to vulnerable groups such as children.

Children's patient data receives enhanced protection under the GDPR[63], particularly in relation to consent, profiling, and digital rights. Profiling children, even with synthetic data, requires a higher standard of care. Although synthetic data may be used to simulate pediatric datasets, the risk of re-identification or wrongful inference remains acute if derived from real-world child data. Therefore, profiling systems trained on synthetic data must be carefully audited to ensure they do not embed or reinforce discriminatory assumptions, especially concerning pediatric populations' developmental, neurological, or genetic conditions. Since the GDPR allows exceptions to ADM and profiling, such uses must include safeguards that protect the child's rights, freedoms, and best interests [64].

In conclusion, while synthetic data holds the potential to support GDPR-compliant innovation in medicine, *it does not automatically neutralize legal risk towards data and privacy protection*.

The increasing use of synthetic data in developing and deploying AI and medical technologies intersects with two additional key legal frameworks in the European Union, alongside the GDPR, notably the AI Act [65] and the MDR[66].

The AI Act introduces a risk-based regulatory framework for AI systems used within the EU[48]. It classifies AI applications into four risk levels (i.e., unacceptable, high, limited, and minimal), with medical AI systems typically categorized as *high-risk* under Annex II. High-risk AI systems, including those used for diagnosis, triage, or treatment recommendations, are subject to stringent transparency, accountability, and human oversight requirements.

The use of synthetic data in training such systems is encouraged, mainly to reduce data protection risks associated with real patient data. However, this does not exempt AI developers from regulatory obligations. If the system is trained on synthetic data but applied to real individuals, it must meet the full spectrum of compliance requirements outlined in the AI Act, ranging from risk and quality management systems [67], data governance and training data requirements [68], transparency and explainability obligations [69], and human oversight mechanisms [70].

Article 10 of the AI Act explicitly requires training, validation, and testing of datasets to be *relevant, representative, error-free, and complete*. This poses a unique challenge for synthetic data, which must reflect real-world diversity and fairness and document the synthetic generation process to ensure that biases or omissions are not unintentionally embedded.

In cases of ADM and profiling that significantly affect individuals, the AI Act reflects GDPR concerns by requiring that users understand how the AI system reaches its decisions[48]. This includes clear documentation of the model's logic, limitations, and the provenance of training data, even if synthetic. The transparency obligation under Article 13 AI Act includes explainability of outputs [71], and where ADM affects children, additional scrutiny is warranted to assess the ethical proportionality of the system's deployment.

The accountability requirement requires developers to ensure traceability of the data lifecycle, from synthetic generation to AI integration. The lack of universal standards for synthetic data governance complicates this requirement, raising the need for independent auditing mechanisms, particularly for systems deployed in clinical environments involving vulnerable groups.

On another page, the MDR governs the rules regarding the placing on the market, making available, or putting into service of medical devices and their accessories for human use within the EU. AI-driven diagnostic tools, decision support systems, and health monitoring platforms using synthetic data typically fall under this regulation when they meet the definition of a medical device [72].

While synthetic data can support pre-market development and post-market surveillance, it introduces distinct challenges to MDR compliance, particularly regarding clinical evidence and performance evaluation [73], cybersecurity and patient safety, risk classification and mitigation strategies [74].

Synthetic data may be used to simulate clinical trials or stress-test device performance. However, when used in place of real-world evidence, regulators must assess whether the synthetic dataset meets the standards for scientific validity, accuracy, and reliability under Annex XIV [75]. This becomes more complicated when children's data is involved, as risk classification may aggravate due to increased vulnerability and potential harm.

Moreover, synthetic data can present cybersecurity risks. Although synthetic datasets are designed to be anonymized, they may still expose system vulnerabilities if exploited during transmission, storage, or third-party processing. The MDR requires manufacturers to ensure state-of-the-art security measures, particularly for AI-enabled devices that process sensitive health data, including that of minors [76].

Children's data raises further concerns. Devices using AI trained on synthetic pediatric data must address specific safety risks, developmental variability, and consent mechanisms. Article 61 MDR emphasizes that clinical investigations involving minors must be scientifically justified and ethically supervised, suggesting that synthetic data use should not bypass ethical review simply because it lacks direct identifiers.

Importantly, MDR requires that post-market surveillance systems [77] monitor device performance, including outcomes derived from synthetic-data-informed models. Any biases, failures, or misclassifications detected during real-world use, particularly those affecting children or underrepresented groups, must be reported and addressed.

In conclusion, patient profiling based on synthetic data presents both an opportunity and a challenge within the AI Act and MDR frameworks. While it can enhance privacy protection and innovation in medicine, *it does not exempt developers or manufacturers from regulatory requirements for accuracy, fairness, explainability, and safety*. Therefore, *synthetic datasets must be traceable, auditable, and technically robust, especially regarding patient profiling applications*.

**Table 2:** Overview of Key Legal Provisions Relevant to Patient Profiling based on Synthetic Data under the GDPR, AI Act, and MDR.

**Legal Framework**	**Relevance**	**Implications**	**Children’s Data**
**GDPR**	*Data protection *Profiling and ADM *Anonymization standards	*Synthetic data *may* fall outside the GDPR scope if fully anonymized (Recital 26) *Risk of re-identification persists *Profiling based on synthetic data may still fall under Article 22 if applied to real individuals	*Children’s data is subject to enhanced protection (Recital 38, Art. 8) *Profiling children, even with synthetic data, requires stricter safeguards
**AI Act**	*Regulation of high-risk *AI Training data standards *ADM transparency and explanation of outcomes	*Synthetic data can be used for training, but must be representative, error-free, and complete (Art. 10) *Traceability and documentation of synthetic generation required *Systems using ADM must be explainable (Art. 13 and 86)	*Use of synthetic pediatric data must address specific safety and consent risks *Clinical justification and ethical oversight required for child-related applications
**MDR**	*Regulation of software and AI as medical devices *Clinical safety *Post-market surveillance	*Synthetic data may support the development, validation, and stress-testing of devices *Not a replacement for clinical evidence unless scientifically validated *Must comply with cybersecurity, traceability, and bias monitoring obligations	*Use of synthetic pediatric data must address specific safety and consent risks *Clinical justification and ethical oversight required for child-related applications

### 2.2 Ethical issues

Using synthetically generated patient data comes with various ethical challenges, especially regarding fairness, autonomy, accuracy, and transparency.

The notion of fairness can be difficult to comprehend since a clear (legal) definition does not exist[78]. It is sometimes associated with non-discrimination frameworks prohibiting unfair discrimination against individuals, for instance, concerning their racial or ethnic origin, sex, age, or disability. Also, the GDPR transposes fairness as one of six data protection principles[79]. Whilst the ethical understanding of fairness can overlap with the concepts embedded in the law (e.g., with the meaning of (non-)discrimination), the former is broader, going beyond the boundaries of the law. Yet, the understanding or perception of what is *fair* remains elusive, particularly because it can differ for every society.

The High-Level Expert Group on AI (HLEG) outlined fairness as an essential principle for trustworthy AI, which consists of two dimensions, namely a substantive and procedural dimension[80]. The substantive dimension reflects the need to ensure an equal distribution of benefits and costs, as well as that individuals and groups are liberated from unfair bias and stigmatisation. This also requires the guarantee of equal opportunities to access such services and technologies and of avoiding any unjustifiable impediment created through AI systems in a person’s decision-making or choice. The procedural element of fairness ensures that individuals can contest decisions generated by AI and the people operating the AI tool[81].

Inaccuracies or biases embedded in AI algorithms may further disadvantage vulnerable groups (e.g., children, persons with disabilities) or populations. AI systems trained on data collected from white populations can significantly impact minorities, possibly leading to detrimental consequences[82]. Besides, power imbalances can lead to unfair outcomes. In healthcare, patients will naturally find themselves in a vulnerable position, seeking medical attention regardless of whether AI is introduced as a mediator[83]; this holds true particularly for pediatric patients. The “AI black-box” aggravates the detection of bias caused by the explainability gap, challenging transparency and trust[84]. *Chauhan et al.* even argue that unbiased datasets are impossible to achieve: not only does data selection and analysis lead to potential bias, but healthcare data is naturally dynamic, which may render certain data outdated over time[85].

Moreover, synthetic data may be prone to misuse when it serves as an alternative for real data. For instance, if care providers or insurance companies use flawed synthetic data to evaluate a possible increase in insurance prices or to draw medical conclusions, this will inevitably come to the detriment of patients, leading to discrimination (e.g., right to access healthcare) and impacting diagnostic outcomes which ultimately deteriorates trust in the use of medical AI[86].

Subsequently, ensuring transparency and accountability is vital to respect pertinent ethical principles (i.e., respect for autonomy, beneficence, non-maleficence, and justice)[87] and to warrant the adequate and safe use of patient profiling systems based on synthetic data. Synthetic data generation models need to be carefully validated and tested, and the output data must be adequately disclosed as synthetic data to avoid being treated as real data[88]. Robust governance frameworks, including data standards and certifications, foster data quality and patient safety[89]. The concept of autonomy relates closely to informed consent, which remains an important instrument to guarantee that individuals are aware of the fact that (and how) their personal data is used for synthetic dataset generation.

**Table 3:** Summary of ethical challenges.

**Ethical principles**	**Relevance**	**Implications**
**Respect for autonomy**	*The principle of respect for autonomy requires ensuring that patients are free in their decision-making process	*Informed consent can be an essential tool in addressing the power imbalance inherent to the patient-care provider relationship
**Principles of Beneficence and Non-maleficence**	*Respecting the principle of beneficence means actively contributing to the health and welfare of patients *The principle of non-maleficence requires refraining from causing harm to patients	*Developers and end-users are required to actively support the health and welfare of patients when creating and applying synthetic data generation techniques *Synthetic data must be of adequate quality, representative, and error-free to prevent harm to patients *Transparency regarding the use of synthetic data and the determination of the purpose for which the synthetic data is generated and used is required
**Principle of Justice**	*The principle of justice entails treating individuals and distributing benefits in a fair, equitable, and appropriate manner	*Synthetic data entails risks when used for the patient’s medical treatment or medical research, possibly leading to discrimination *Must prevent an impediment to the individual’s right to access healthcare (procedural fairness)

## 3 Conclusion

Synthetic data has emerged as a powerful enabler of innovation in healthcare, particularly by supporting privacy-preserving patient profiling. By simulating the statistical characteristics of real datasets without revealing identifiable information, fully synthetic data offers a promising response to longstanding privacy concerns and barriers to data access in healthcare research and AI development. However, this paper has shown that synthetic data is not a regulatory or ethical panacea.

Drawing on a stratified taxonomy, distinguishing fully synthetic, partially synthetic, and hybrid synthetic data, this paper has demonstrated that only certain forms of synthetic data may fall outside the scope of the GDPR, depending on how they are generated and used. While often privacy-enhancing, profiling systems based on synthetic data may produce outputs that raise legal and ethical concerns when applied to real individuals, especially in high-risk contexts regulated by the GDPR, AI Act, and Medical Devices Regulation.

From an ethical standpoint, the four biomedical principles, such as autonomy, beneficence, non-maleficence, and justice, remain essential to evaluating the fairness and transparency of profiling systems built on synthetic data. Even when personal data is not directly involved, the potential for bias, opacity, or unintended harm demands critical scrutiny. This paper ultimately calls for a context-sensitive legal and ethical framework that distinguishes between different types and uses of synthetic data. To responsibly unlock the benefits of synthetic data in patient profiling, it is crucial to establish rigorous accountability mechanisms, generation standards, and domain-specific guidelines. Accomplishing this will support innovation and scalability in digital medicine, trust, compliance, and safeguarding fundamental rights.

## CRediT authorship contribution statement

**Daniela Spajic:** Writing – original draft. **Dusko Milojevic:** Writing – review & editing, Writing – original draft. **Maja Nisevic:** Writing – review & editing, Writing – original draft.

## Ethical approval

This article does not contain any studies with human participants or animals performed by any of the authors.

## Declaration of Competing Interest

The authors declare that they have no known competing financial interests or personal relationships that could have appeared to influence the work reported in this paper.
